# Safety assessment of a redirection program using an electronic application for low-acuity patients visiting an emergency department

**DOI:** 10.1186/s12873-022-00626-4

**Published:** 2022-04-29

**Authors:** Anne-Laure Feral-Pierssens, Judy Morris, Martin Marquis, Raoul Daoust, Alexis Cournoyer, Justine Lessard, Simon Berthelot, Alexandre Messier

**Affiliations:** 1 Hôpital du Sacré-Cœur de Montréal, CIUSSS-NIM, 5400 boulevard Gouin Ouest, Montréal, Québec H4J 1C5 Canada; 2 CR-CSIS, Sherbrooke University, Longueuil, Québec Canada; 3 Health Educations and Promotion Laboratory (LEPS EA3412), University Sorbonne Paris Nord, Bobigny, France; 4 SAMU 93 - Emergency Department, Avicenne Hospital, Assistance Publique Hôpitaux de Paris, Bobigny, France; 5 Département médecine de famille et médecine d’urgence, Université de Montréal, Montréal, Québec Canada; 6 Hôpital Maisonneuve-Rosemont, CIUSSS-EIM, Montréal, Québec Canada; 7 Corporation d’Urgences-santé, Montréal, Québec Canada; 8 Département de médecine familiale et de médecine d’urgence, Université Laval, Québec, Canada; 9 Axe Santé des populations et Pratiques optimales en santé, Centre de recherche du CHU de Québec-Université Laval, Québec, Canada

**Keywords:** Redirection, Low-acuity patients, Healthcare Use, Quality of care

## Abstract

**Background:**

Emergency departments (EDs) are operating at or above capacity, which has negative consequences on patients in terms of quality of care and morbi-mortality. Redirection strategies for low-acuity ED patients to primary care practices are usually based on subjective eligibility criteria that sometimes necessitate formal medical assessment. Literature investigating the effect of those interventions is equivocal. The aim of the present study was to assess the safety of a redirection process using an electronic clinical support system used by the triage nurse without physician assessment.

**Methods:**

A single cohort observational study was performed in the ED of a level 1 academic trauma center. All low-acuity patients redirected to nearby clinics through a clinical decision support system (February–August 2017) were included. This system uses different sets of medical prerequisites to identify patients eligible to redirection. Data on safety and patient experience were collected through phone questionnaires on day 2 and 10 after ED visit. The primary endpoint was the rate of redirected patients returning to any ED for an unexpected visit within 48 h. Secondary endpoints were the incidence of 7-day return visit and satisfaction rates.

**Results:**

A total of 980 redirected low-acuity patients were included over the period: 18 patients (2.8%) returned unexpectedly to an ED within 48 h and 31 patients (4.8%) within 7 days. No hospital admission or death were reported within 7 days following the first ED visit. Among redirected patients, 81% were satisfied with care provided by the clinic staff.

**Conclusion:**

The implementation of a specific electronic-guided decision support redirection protocol appeared to provide safe deferral to nearby clinics for redirected low-acuity patients. EDs are pivotal elements of the healthcare system pathway and redirection process could represent an interesting tool to improve the care to low-acuity patients.

## Background

Emergency Departments (ED) are pivotal elements of the healthcare system pathway. They are sensitive to patient flow and can be impacted by the accessibility and the use of primary care facilities on the one hand and by hospital capacities on the other hand. In many developed countries, EDs are operating at or above capacity, facing the same phenomenon of “overcrowding” which is often due to downstream congestion with ED patients waiting for a bed on hospital wards and also to the ever-increasing number of ED visits [1–4]. This phenomenon has significant consequences on the ease of access to ED care; with high rate of patients leaving without being seen (LWBS) by an emergency physician, suboptimal quality of care, higher morbi-mortality and even altered quality of life at work for ED staff [5–9].

To address this complex issue, ED flow management strategies have focused on some possible solutions, one of which is the identification of low-acuity patients that could be taken care of in other medical settings or in specific fast tracks, hoping to reduce ED workload and shortening patients’ length-of-stay. EDs have elaborated different interventions addressing this issue with on-site or remote redirection process [10–17]. These interventions have been implemented to pursue different objectives among which to provide appropriate care to low-acuity patients (avoiding suboptimal care, over prescription and over diagnostic) and to concentrate the main ED resources for patients needing emergency care. However, literature investigating the effect of those interventions is equivocal [18]. While some authors report an improvement of ED flow indicators, high patients’ satisfaction and low rates of unexpected ED return visits [13–15, 19, 20], other authors report an increase hospital admission rate 7 days after redirection and no impact on ED indicators [21]. These conflicting results have led to controversy over the potential impact and safety of such interventions [22, 23].

Correctly identifying patients that could benefit from this type of intervention and providing them with a safe healthcare pathway remain the cornerstone of the redirection process. Indeed, the ideal identification strategy should be able to select the majority of patients that could follow the redirection track without jeopardizing their health status. Since there is little consensus on the definition of low-acuity patients [24–31], ED triaging is one of the main tools reported in literature to identify such patients. However, ED triage is partially based on immediate vital risk assessment, it has not been designed for selecting patients eligible to redirection [32]. The triaging process and its interrater reliability can also be impacted by other determinants such as nurse training or the use of electronic clinical decision support system [33–38]. Other strategies are used to identify redirection eligible patients such as implementing a systematic emergency physician assessment at the ED entrance or selecting them based on their chief complaint. Both strategies have important limits and are not reproducible nor can they be extrapolated to other settings [21, 39, 40]. A proper identification strategy should take into account various patient information, medical history, vital risk assessment, chief complaint, but also its environment and comprehensiveness of the redirection process. Patients eligible to redirection should be identified quickly upon ED presentation and they should be offered a precise appointment with a general physician (GP). This process should also be reproducible and transposable. The use of an electronic application could improve these characteristics over subjective and manual process.

When introducing a new healthcare pathway, patient safety should also be assessed. Safety is a component of quality of care and is usually defined as avoiding or mitigating unintended injuries from the delivery of health care [41]. In the ED, aside from the onset of adverse events, unscheduled return visit within a few days following a first visit is considered as an important indicator of the quality of care provided in the emergency settings [42, 43]. Thus, this safety indicator could be assessed among redirected patients to analyze further these different healthcare pathways.

As such, the aim of the present study was to assess the safety of a redirection process of low-acuity ED patients to a nearby clinic using an electronic clinical support system that helps patient identification and appointment scheduling.

## Methods

### Settings and study design

The present study is a monocentric single cohort observational study of professional practices focused on redirected patients which were recruited between February 14th and August 17th 2017. It was approved by the local institutional review and research ethical board. Informed oral consent was obtained when the patients were called for follow-up, parents’ oral consent was obtained for minor patients.

**Fig. 1:**
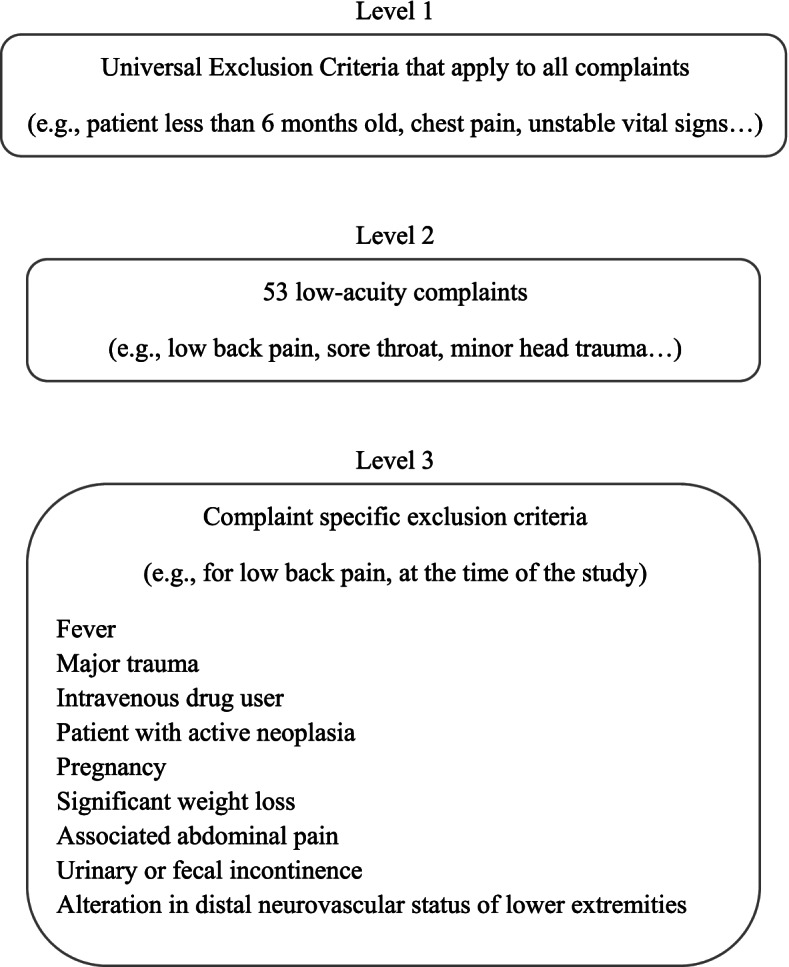
Example of the clinical decision support system using different sets of medical prerequisites to help identify low-acuity patients who could be redirected to nearby medical clinics

The study was conducted in the ED of a level 1 trauma center and academic hospital with an annual census of approximately 65,000 mostly adult ED visits. In 2015, a redirection program for low-acuity patients was implemented following the development of a local algorithm to select these patients. It was developed through collaborative work and joint reflection between ED physicians, triage nurses, and the associated clinics’ practicing GPs. This clinical decision support uses different chief complaints, sets of medical prerequisites and contraindications to help identify patients who can be redirected (Fig. 1). Patients eligible to redirection are selected based on their chief complaint which is collected by the nurse at triage (e.g: sore throat, low back pain, minor head trauma). The redirection process can only be performed after the usual first triage step has been fully completed using the CTAS as a specific electronic clinical decision support system to assign a triage level to the patient. Then, if the nurse raises the possibility that the patient could be redirected to another healthcare provider, she consults the electronic clinical decision support system dedicated to redirection to clear any contra indications. Two levels of contra indications must be checked. First, universal contra indications must be ruled out regardless of the main complaint such as abnormal vital signs or patients age (≤6 months old). Second, the nurse must verify that the patient does not present any specific contra indications associated to his main complaint (Fig. 1). To ease the use of this algorithm and allow, at the same time, to schedule an appointment to one of the three nearby medical clinics (situated within 5 km from the hospital), an electronic tool has been designed. Therefore, this application, which includes the algorithm, does not provide any specific diagnosis and is not a substitute for medical assessment. In summary, triage nurses using the electronic tool can, following a verification process, decide if a particular patient is to be redirected to a nearby clinic or not. Redirection was offered to eligible patients but was not imposed. Appointments were scheduled on the same day if possible or the next day at most.

### Selection of Participants

**Fig. 2:**
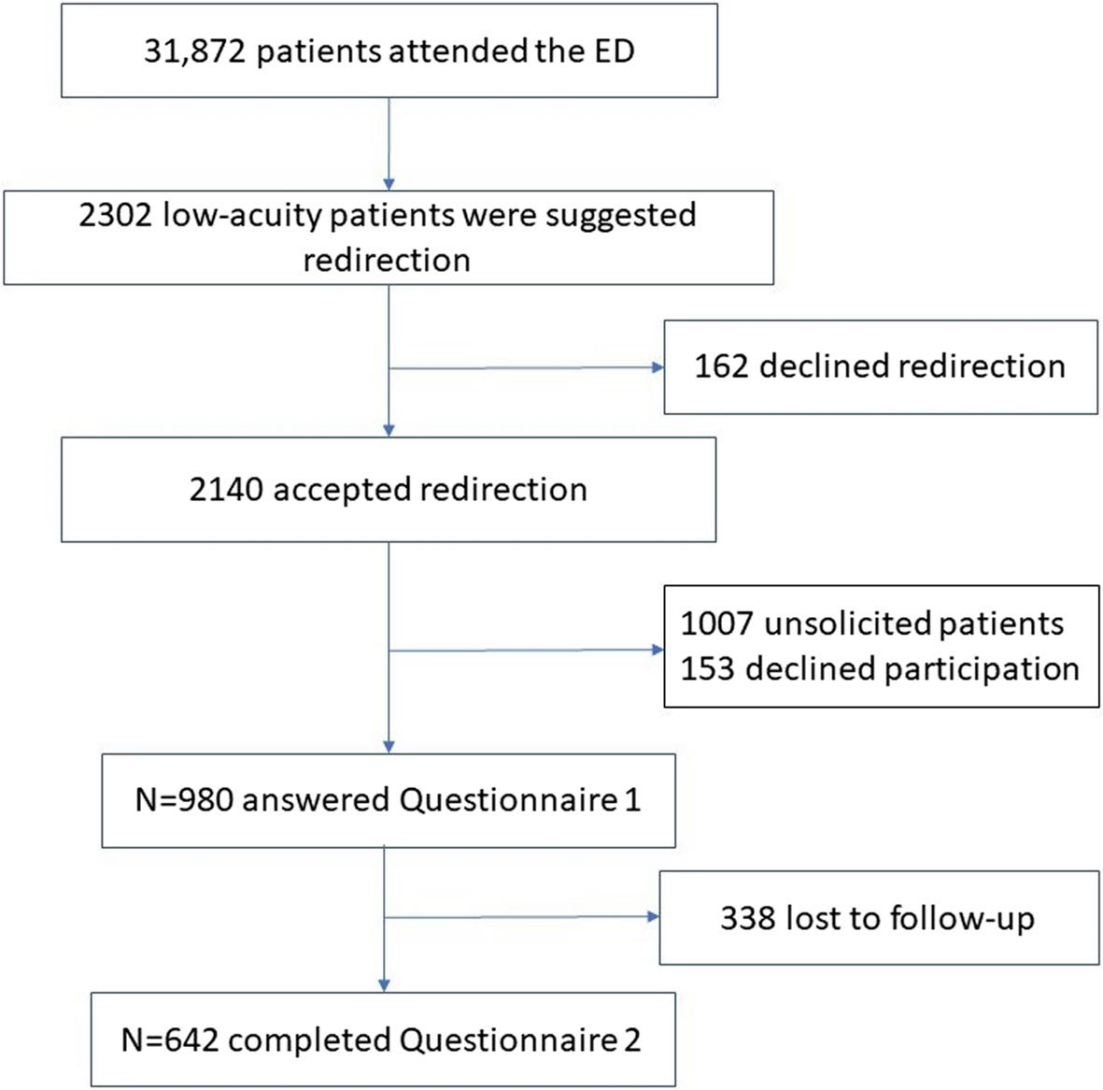
Flow chart

Patients eligible to redirection after being screened by the triage nurse relying on the pre-specified algorithm were considered for inclusion. Low-acuity ED patients who accepted to be redirected were prospectively recruited from February 14 to August 17, 2017 among patients that had been offered redirection (Fig. 2). We excluded patients that were not able to speak French or English.

### Data collection

Demographic and clinical characteristics of the included patients were collected from electronic medical records. Outcome data were collected through questionnaires administered at 2 follow-up phone calls. The first one was administered within 48 h after ED presentation while the second was administered 7 to 10 days after the initial ED visit depending on patients’ availability. Questionnaires included information about the patient’s experience through the process and redirection safety issues. Unexpected returns to any healthcare facility were collected. Five-levels Likert scale questions were used to assess patient’s satisfaction. For patients lost to follow up between Questionnaire 1 and Questionnaire 2, the ED electronic medical records were reviewed for any new ED visit in the same hospital.

### Outcomes

The primary outcome was the rate of redirected patients returning to any ED for an unexpected visit within 48 h after the first ED visit.

The secondary outcomes were 1) the rate of redirected patients returning to any hospital for an unexpected visit within 7 days after the first ED visit 2) the rate of patients satisfied with their redirection experience.

### Data analysis

Continuous data were expressed as mean (standard deviation) if normally distributed or median (interquartile range) if not. Categorical data were reported as number and percentage (95% confidence interval). Answers to patient satisfaction questionnaires were analyzed using descriptive statistics. For each question, the rate of satisfied/very satisfied, neutral, dissatisfied/very dissatisfied patients were presented as means with 95% confidence intervals. Stratified analysis were also performed depending on triage priority and age category (pediatric vs adult). All analysis were conducted using SPSS version 23.

## Results

During the study period, 2140 low-acuity patients visited the ED and accepted to be redirected after triage (6.7% of all visiting patients and 15.3% of ambulatory patients). Among them, 980 patients were included in the study and answered the first questionnaire and 642 (65.5%) also answered the second questionnaire.

Baseline characteristics of included patients are presented in Table 1 along with the characteristics of all patients who accepted and refused redirection after nurse triage. Mean age for patients of our sample answering Questionnaire 1 was 42 (28–56) years old (y.o), with 61 patients (6.2%) less than 18 yo. and 81.9% were categorized through triage as priority Level 4 or 5. Almost all redirected patients (94.1%; 95% CI 92.4–95.5) attended their medical clinic appointment.

**Table 1:** Baseline characteristics of redirected patients over the period study and included patients

Characteristics	Accepting redirection (***n*** = 2140)	Questionnaire 1 Patients (***n*** = 980)
	**N(%)**	**N(%)**
Age (years), median (Q1;Q3)	38 (23–54)	40 (28–56)
Pediatric case	253 (11.8)	59 (6.0)
Male	1095 (51.2)	502 (51.2)
Triage priority		
Level 1	0 (0)	0 (0)
Level 2	21 (1.0)	11 (1.1)
Level 3	330 (15.4)	167 (17.0)
Level 4	1039 (48.6)	469 (47.9)
Level 5	749 (35.0)	333 (34.0)
		***n*** **= 939**
Transportation mode		
Car		647 (68.9)
Taxi		4 (5.1)
Public transportation		86 (9.2)
Other		158 (16.8)
Accompanied to the ED		472 (50.2)
Called GP before the ED visit		
Yes		145 (15.4)
No appointed GP		293 (31.2)

*ED* Emergency Department, *GP* General Physician

### Patient Safety

Out of all patients that were redirected, attended their clinic appointment, and completed Questionnaire 2 (*n* = 642), 18 patients (2.8%) returned unexpectedly to ED within 48 h after their initial ED visit (Table 2). It concerned 2.7% of adult patients and 4.8% of pediatric patients. Among patients with Canadian Emergency Department Triage and Acuity Scale (CTAS) 3, 4 or 5 score, 3.7, 2.6 and 2.7% returned unexpectedly to ED within 48 h, respectively. Within 7 days following ED visit, 31 patients (4.8%) returned unexpectedly to the ED (29 for adults (4.8%) and 2 for children (4.8%) (Table 3). Those unexpected ED visits concerned 7.3, 3.6 and 5.4% of patients that had CTAS 3, 4, and 5 score, respectively. Patients who returned unexpectedly to any healthcare facility were mostly complaining of limb muskuloskeletal pain or reporting dermatology issues. There was no difference in the distribution of complaint’s categories between patients who returned unexpectedly and patients that did not. No hospital admission or death was reported within 7 days following the first ED visit. For patients that were lost to follow-up between their first and their second questionnaire (338 patients), 10 (2.96%) of them visited the same ED within 48 h of their initial ED visit and 18 patients (5.3%) within 7 days.

**Table 2:** Rate of unexpected returns to a healthcare facility within 48 h following the ED visit and after redirection process

	Patients n/total (%)
**For all redirected patients**	
• Unexpected returns to any hospital, n (%)	18/642 (2.8)
• Unexpected returns to the same clinic, n (%)	5/642 (0.8)
• Unexpected returns to any type of healthcare facility, n (%)	30/642 (4.7)
**For adult patients only**	
• Unexpected returns to any hospital, n (%)	16/600 (2.7)
• Unexpected returns to the same clinic, n	5/600 (0.8)
• Unexpected returns to any type of healthcare facility, n (%)	27/600 (4.5)
**For pediatric patients only**	
• Unexpected returns to any hospital, n (%)	2/42 (4.8)
• Unexpected returns to the same clinic, n	0/42 (0)
• Unexpected returns to any type of healthcare facility, n (%)	3/42 (7.1)
**For patients triaged Level 3**	
• Unexpected returns to any hospital, n (%)	4/109 (3.7)
• Unexpected returns to the same clinic, n	0/109 (0)
• Unexpected returns to any type of healthcare facility, n (%)	8/109 (7.3)
**For patients triaged Level 4**	
• Unexpected returns to any hospital, n (%)	8/305 (2.6)
• Unexpected returns to the same clinic, n	4/305 (1.3)
• Unexpected returns to any type of healthcare facility, n (%)	13/305 (4.3)
**For patients triaged Level 5**	
• Unexpected returns to any hospital, n (%)	6/223 (2.7)
• Unexpected returns to the same clinic, n	1/223 (0.4)
• Unexpected returns to any type of healthcare facility, n (%)	9/223 (4.0)

**Table 3:** Rate of unexpected returns to a healthcare facility within 7 days following the ED visit and after redirection process

	Patients n/total (%)
**For all redirected patients**	
• Unexpected returns to any hospital	31/642 (4.8)
• Unexpected returns to the same clinic	13/642 (2.0)
• Unexpected returns to any type of healthcare facility, n (%)	62/642 (9.7)
**For adult patients only**	
• Unexpected returns to any hospital	29/600 (4.8)
• Unexpected returns to the same clinic	13/600 (2.2)
• Unexpected returns to any type of healthcare facility, n (%)	58/600 (9.7)
**For pediatric patients only**	
• Unexpected returns to any hospital	2/42 (4.8)
• Unexpected returns to the same clinic	0/42 (0)
• Unexpected returns to any type of healthcare facility, n (%)	5/42 (11.9)
**For patients triaged Level 3**	
• Unexpected returns to any hospital	8/109 (7.3)
• Unexpected returns to the same clinic	1/109 (0.9)
• Unexpected returns to any type of healthcare facility, n (%)	17/109 (15.6)
**For patients triaged Level 4**	
Unexpected returns to any hospital	11/305 (3.6)
Unexpected returns to the same clinic	7/305 (2.3)
Unexpected returns to any type of healthcare facility, n (%)	23/305 (7.5)
**For patients triaged Level 5**	
Unexpected returns to any hospital	12/223 (5.4)
Unexpected returns to the same clinic	5/223 (2.2)
Unexpected returns to any type of healthcare facility, n (%)	22/223 (9.9)

### Patient experience

Among all patients who went through the redirection process, the overall satisfaction rate (as very satisfied or satisfied) was 84%. Patients’ satisfaction with the suggested appointment and clinic availabilities are presented in Table 4. Among redirected patients, 94% of them reported to have gone to their clinic appointment and 81% of those patients were satisfied with care provided by the clinic staff. Finally, 92% of redirected patients stated they would consider the redirection process for future ED visit.

**Table 4:** Rate of patients reporting to be satisfied or very satisfied over the redirection process

Steps of the redirection process	All patients n/total (%)	Adults n/total (%)	Children n/total (%) ***N*** = 61	Level 3 n/total (%) ***N*** = 167	Level 4 n/total (%) ***N*** = 469	Level 5 n/total (%) ***N*** = 333
Redirection Suggestion by the triage nurse	830/931 (89)	778/874 (89)	52/57 (91)	137/159 (86)	396/444 (89)	291/318 (92)
Explanations given by the triage nurse	826/928 (89)	773/871 (89)	53/57 (93)	139/158 (88)	395/442 (89)	285/318 (90)
Days availability for clinic appointment	882/924 (96)	826/867 (95)	56/57 (98)	151/158 (96)	420/439 (96)	302/317 (95)
Time slots availability for clinic appointment	859/923 (93)	806/866 (93)	53/57 (93)	140/158 (89)	418/438 (95)	293/317 (92)
Overall satisfaction with the redirection process	779/923 (84)	730/865 (84)	49/58 (84)	124/158 (79)	373/438 (85)	275/317 (87)
Would consider the redirection process in the future	841/916 (92)	786/859 (92)	55/57 (96)	142/157 (90)	400/435 (92)	290/314 (92)
The redirection process should be considered at provincial level	871/909 (96)	815/851 (96)	56/58 (97)	149/158 (94)	412/427 (96)	301/314 (96)
Patient present at the appointment	874/929 (94)	821/870 (94)	53/59 (90)	150/159 (94)	415/443 (94)	299/317 (94)
Care provided at the clinic	699/865 (81)	656/814 (81)	43/51 (84)	111/148 (75)	347/409 (85)	234/298 (79)

## Discussion

This study investigating the impact of a redirection process of low-acuity ED patients to nearby clinics using an electronic clinical decision support system showed a low rate of unexpected returns to any ED two and seven days after the first visit, and no hospital admission. Almost 7% of all ED visits and 15% of ambulatory visits have been redirected. The satisfaction rates of these patients were high.

As redirection strategies differ, comparisons with the existing literature on the subjects is difficult [18, 44]. Some authors across different healthcare systems investigated the rate of unexpected return visit to the ED for all ED patients. They reported similar or higher return visit rates (2 to 5%) than the one we report for redirected patients [42, 43, 45, 46]. Murphy et al. who performed a randomized controlled trial (testing the redirection of low-acuity patients to a GP vs usual ED care) reported similar rates of unexpected returns in both pathways [14]. Their rate of return within a month of the first visit was however much higher (~17%) than in the present study. He also reported other outcomes such as fewer investigations and admissions and higher prescriptions for patients managed by GPs. This could be explained by the heterogeneity of included patients since 66% of all ED visits were considered eligible for the trial. Bentley et al. observed in their cohort study that 6% of redirected patients were admitted 7 days after the first ED visit. Their intervention involved physician assessment at triage and medical decision to redirect patients [21]. The strategy that has been investigated in the present study, which is based on chief complaint’s selection, seems reassuring. It can be hypothesized that the use of an electronic clinical decision support system contributed to respect the eligibility and contraindications to redirection, which might help in preventing adverse outcomes for redirected patients.

The main outcome of the present study was the rate of patients returning to any ED within 48 h following the first ED visit rather than seven days. The 7-day return rate may be a less relevant indicator than the 48 h rate when investigating the safety of a redirection process. Indeed, among all patients presenting with an acute disease, illness progression is always possible despite an efficient first medical assessment and appropriate treatment (in the ED or in a clinic). Depending on the pathology, the worsening of an acute disease may be seen or reported a few days after the first medical assessment and could end up in a second ED visit a week later. Literature focusing on unscheduled return visits observed that most of these visits are due to the illness progression and patient non-compliance to treatment rather than medical errors [45, 47].

Redirection programs must be safe for ED patients but they must have also a sufficient impact to justify their deployment and sustainability. This sensitive relationship between those two outcomes is the main determinant of the intervention success or failure. Previous publications on redirection strategies report a redirection rate among ambulatory ED patients ranging from 2 to 20% [18, 40, 47]. The redirection rate depends on many factors such as the determinants and criteria used for the selection process of eligible patients (which echoes the many definitions of low-acuity patients), the ED staff training and its confidence in the process, the reactivity and availability of collaborating clinics and the accessibility of the suggested pathway for redirected patient. The variability of the different determinants and inputs of a redirection process can explain the heterogeneity of the literature reporting this indicator [18]. We reported a redirection rate of 6.7% of all visiting patients and 15.3% of ambulatory patients. In this study, the selection process of eligible patients relies on an electronic clinical support system. This particularity might increase the reproducibility of the results. Future studies focusing on the direct impact of the redirection process on ED flow and performance indicators would be interesting and complementary. A prospective multicentric study would compare on the one hand, the health care use, consumption and pathways of low-acuity patients whether redirected or not and would assess the variation of ED indicators following the implementation process on the other hand.

## Limitations

A large proportion of patients were not solicited for recruitment due to the availability of research staff, which led to the usage of a convenience sample and limits results from being transposable. However, the sample included had similar demographic characteristics and triage categories as the overall eligible population (Table 1). Some patients were also lost to follow-up between their first and their second questionnaire. However, for these patients, the ED electronic medical records were searched for return visits in the same hospital as their index visit. The 48 h and 7 days return rates were similar to those of the included patients still underestimation can not be excluded since they could also have consulted in a different ED. The present study did not provide comparisons with a control group such as patients eligible to redirection but refusing to process and rather staying in the conventional ED pathway. Since we based the redirection process on a panel of medical and social characteristics that are not usually collected in the electronic medical charts, we could not easily and precisely identify a control group through retrospective methodology. Our present study was focused on the evaluation of professional practices and health trajectory of redirected patients. Further studies should focus on a prospective and comparative analysis between low-acuity ED patients taken care of in the usual settings and those selected for redirection. This study is focused on an academic hospital in a dense territory which can limits extrapolation of our results to other settings such as suburban or rural hospitals with different incoming ED populations and various organization of healthcare providers such as farther collaborating clinics. Finally, this study is investigating the safety of a redirection process using a specific electronic clinical support system which helps identifying eligible low-acuity patients, thus its results cannot be transposed to other redirection protocols with different low-acuity patients definitions.

## Conclusion

The study reported here investigates the safety of a redirection process of low-acuity ED patients to nearby clinics using an electronic clinical support system. The results showed a low rate of unexpected visit within 48 h and 7 days. Satisfaction rates were high amongst redirected patients. Emergency Departments (ED) are pivotal elements of the healthcare system pathway and redirection process could represent an interesting tool to improve the care to low-acuity patients.

## Data Availability

The data underlying this article cannot be shared publicly due to federal and provincial legislations protecting personal data and materials in Canada and Quebec. Access to data and material can be provided upon request to the corresponding author.

## Abbreviations

CTAS: Canadian Emergency Department Triage and Acuity Scale
ED: Emergency Departments
GP: General Physician
LWBS: Left Without Being Seen

## References

[1] Affleck A, Parks P, Drummond A, Rowe BH, Ovens HJ (2013). Emergency department overcrowding and access block. CJEM..

[2] Goodell S, DeLia D, Cantor JC. Emergency Department Utilization and Capacity. Robert Wood Johnson Foundation. 2009; [Accesses November 22nd 2020]. Available: https://www.rwjf.org/en/library/research/2009/07/emergency-department-utilization-and-capacity0.html.

[3] Hoot NR, Aronsky D (2008). Systematic review of emergency department crowding: causes, effects, and solutions. Ann Emerg Med.

[4] Morley C, Unwin M, Peterson GM, Stankovich J, Kinsman L (2018). Emergency department crowding: A systematic review of causes, consequences and solutions. PLoS One.

[5] Guttman A, Schull M, Vermeulen M, Stukel T (2011). Association between waiting times and short term mortality and hospital admission after departure from emergency department: population based cohort study from Ontario, Canada. BMJ.

[6] Thibon E, Bobbia X, Blanchard B (2019). Association between Mortality and Waiting Time in Emergency Room among Adults Hospitalized for Medical Etiologies. Annales Fr Med Urg.

[7] Johnston A, Abraham L, Greenslade J (2016). Review article: Staff perception of the emergency department working environment: Integrative review of the literature. Emerg Med Australas.

[8] Javidan AP, Hansen K, Higginson I, Jones P, Lang E (2021). The International federation for emergency medicine report on emergency department crowding and access block: a brief summary. CJEM.

[9] Clouston R, Atkinson P, Canales DD, Fraser J, Sohi D, Lee S, et al. Emergency department occupancy is useful as a simple real-time measure of crowding. CJEM. 2021. 10.1007/s43678-021-00098-8. 10.1007/s43678-021-00098-8 33748940

[10] Cooper A, Edwards M, Brandling J (2019). Taxonomy of the form and function of primary care services in or alongside emergency departments: concepts paper. Emerg Med J.

[11] Cooper A, Davies F, Edwards M (2019). The impact of general practitioners working in or alongside emergency departments: a rapid realist review. BMJ Open.

[12] Ramlakhan S, Mason S, O’Keeffe C, Ramtahal A, Ablard S (2016). Primary care services located with EDs: a review of effectiveness. Emerg Med J.

[13] Sharma A, Inder B (2011). Impact of co-located general practitioner (GP) clinics and patient choice on duration of wait in the emergency department. Emerg Med J.

[14] Murphy AW, Bury G, Plunkett PK (1996). Randomised controlled trial of general practitioner versus usual medical care in an urban accident and emergency department: process, outcome, and comparative cost. BMJ.

[15] Boeke AJP, van Randwijck-Jacobze ME, de Lange-Klerk EM, Grol SM, Kramer MH, van der Horst HE (2010). Effectiveness of GPs in accident and emergency departments. Br J Gen Pract.

[16] Wang M, Wild S, Hilfiker G (2014). Hospital-integrated general practice: a promising way to manage walk-in patients in emergency departments. J Eval Clin Pract.

[17] Chrusciel J, Fontaine X, Devillard A, Cordonnier A (2019). Impact of the implementation of a fast-track on emergency department length of stay and quality of care indicators in the Champagne-Ardenne region: a before-after study. BMJ Open.

[18] Gonçalves-Bradley D, Khangura JK, Flodgren G, Perera R, Rowe BH, Shepperd S (2018). Primary care professionals providing non-urgent care in hospital emergency departments. Cochrane Database Syst Rev.

[19] Rodi SW, Grau MV, Orsini CM (2006). Evaluation of a fast track unit: alignment of resources and demand results in improved satisfaction and decreased length of stay for emergency department patients. Qual Manag Health Care.

[20] Wiler JL, Gentle C, Halfpenny JM (2010). Optimizing emergency department front-end operations. Ann Emerg Med.

[21] Bentley JA, Thakore S, Morrison W, Wang W (2017). Emergency Department redirection to primary care: a prospective evaluation of practice. Scott Med J.

[22] Berthelot S, Lang ES, Messier A (2020). CJEM Debate Series: #EDRedirection - Sending low-acuity patients away from the emergency department - An imperative for appropriateness and integration. CJEM.

[23] Rowe BH, Ovens H, Schull MJ (2020). CJEM Debate Series: #EDRedirection - Efforts to divert patients from the emergency department - Stop blaming the patients! An argument against redirection. CJEM.

[24] Naouri D, Ranchon G, Vuagnat A (2019). Factors associated with inappropriate use of emergency departments: findings from a cross-sectional national study in France. BMJ Qual Saf.

[25] Nagree Y, Ercleve TNO, Sprivulis PC (2004). After-hours general practice clinics are unlikely to reduce low acuity patient attendances to metropolitan Perth emergency departments. Aust Health Rev.

[26] Uscher-Pines L. Applying What Works to Reduce Non-Urgent Emergency Department Use. 2013 [Accessed september 19th 2020]. Available: https://www.rand.org/blog/2013/05/applying-what-works-to-reduce-non-urgent-emergency.html

[27] Afilalo J, Marinovich A, Afilalo M (2004). Nonurgent Emergency Department Patient Characteristics and Barriers to Primary Care. Acad Emerg Med.

[28] Anantharaman V (2008). Impact of health care system interventions on emergency department utilization and overcrowding in Singapore. Int J Emerg Med.

[29] Arain M, Campbell MJ, Nicholl JP (2015). Impact of a GP-led walk-in centre on NHS emergency departments. Emerg Med J.

[30] Buckley DJ, Curtis PW, McGirr JG (2010). The effect of a general practice after-hours clinic on emergency department presentations: a regression time series analysis. Health Care.

[31] Whittaker W, Anselmi L, Kristensen SR (2016). Associations between extending access to primary care and emergency department visits: a difference-in-differences analysis. PLoS Med.

[32] Vertesi L (2004). Does the Canadian Emergency Department Triage and Acuity Scale identify non-urgent patients who can be triaged away from the emergency department?. CJEM.

[33] Mrihaghi A, Heydari A, Mazlom R, Ebrahimi M (2015). The reliability of the Canadian Triage and Acuity Scale: Meta-Analysis. N Am J Med Sci.

[34] Gravel J, Gouin S, Goldman RD, Osmond M (2012). The Canadian Triage and Acuity Scale for Children: a prospective multicenter evaluation. Ann Emerg Med.

[35] Dong SL, Bullard MJ, Meurer D, Blitz S, Holroyd BR, Rowe BH (2007). The effect of training on nurse agreement using an electronic triage system. CJEM.

[36] Grafstein E, Innes G, Westman J, Christenson J, Thorne A (2003). Inter-rater reliability of a computerized presenting complaint linked triage system in an urban emergency department. CJEM.

[37] Dallaire C, Poitras J, Aubin K, Lavoie A, Moore L (2012). Emergency department triage: Do experienced nurses agree on triage scores.?. J Emerg Med..

[38] Manos D, Petrie DA, Beveridge RC, Walter S, Ducharme J (2002). Inter-observer agreement using the Canadian emergency department triage and acuity scale. CJEM.

[39] Khangura JK, Flodgren G, Perera R, Rowe BH, Shepperd S (2012). Primary care professionals providing non-urgent care in hospital emergency departments. Cochrane Database Syst Rev.

[40] Morin C, Choukroun J, Callahan J-C (2018). Safety and efficiency of a redirection procedure toward an out of hours general practice before admission to an emergency department, an observational study. BMC Emerg Med.

[41] Dy S, Gurses AP (2010). Care pathways and patient safety: key concepts, patient outcomes and related interventions. Int J Care Pathways.

[42] Sauvin G, Freund Y, Saidi K, Riou B, Hausfater P (2013). Unscheduled return visits to the emergency department: consequences for triage. Acad Emerg Med.

[43] Pereira L, Choquet C, Perozziello A, Wargon M, Juillien G, Colosi L, Hellmann R, Ranaivoson M, Casalino E (2015). Unscheduled-return-visits after an emergency department (ED) attendance and clinical link between both visits in patients aged 75 years and over: a prospective observational study. PLoS One.

[44] Kirkland SW, Soleimani A, Rowe BH, Newton AS (2019). A systematic review examining the impact of redirecting low-acuity patients seeking emergency department care: is the juice worth the squeeze?. Emerg Med J.

[45] Wu CL, Wang FT, Chiang YC, Lin TG, Fu LF, Tsai TL (2010). Unplanned emergency department revisists with 72 hours to a secondary teaching referral hospital in Taiwan. J Emerg Med.

[46] Cheng SY, Wang HT, Lee CW, Tsai TC, Hung CW, Wu KH (2013). The characteristics and prognostic predictors of unplanned hospital admission within 72 hours after ED discharge. Am J Emerg Med.

[47] Thijssen W, Wijnen-van Houts M, Koetsenruijter J, Giesen P, Wensing M (2013). The impact on emergency department utilization and patient flows after integrating with a general practitioner cooperative: an observational study. Emerg Med Int.

